# New York

**Published:** 1896-05

**Authors:** 


*New York.* The *Tribune* of New York City, in a recent editorial referring to the bill destined to provide one million dollars for controlling and eradicating bovine tuberculosis, thought it a very modest sum under all the circumstances, and in commending the appropriation thought that one should not be long in deciding such a question when the State annually lost 5000 to 6000 of her people from this disease, and that one very great source of its existence, propagation, and perpetuation could be largely removed by this sum, which, when compared with this great loss, paled into insignificance. The *Tribune* favorably commends the passage of the bill, and well asks, What would the people of the Empire State not do if a similar number of people died in any one year from any other disease?

Another bill has been presented in the Assembly to reopen the registration-books for non-graduates for a period of thirty days, and to admit to registration those who had been in practice for three years preceding the passage of the original act. This looks like another effort to allow a single individual to register and thus avoid the criticism of special legislation. The committee is alive to its existence, and will no doubt check it before it reaches a place on the calendar. When will these efforts to evade the law cease?

